# Gratitude at Work Prospectively Predicts Lower Workplace Materialism: A Three-Wave Longitudinal Study in Chile

**DOI:** 10.3390/ijerph18073787

**Published:** 2021-04-05

**Authors:** Jesús Unanue, Xavier Oriol, Juan Carlos Oyanedel, Andrés Rubio, Wenceslao Unanue

**Affiliations:** 1 Programa de Doctorado en Educación y Sociedad, Facultad de Educación y Ciencias Sociales, Universidad Andres Bello, Santiago 7550000, Chile; 2 Research Institute on Quality of Life, University of Girona, 17004 Girona, Spain; xavier.oriol@udg.edu; 3 Facultad de Educación y Ciencias Sociales, Universidad Andres Bello, Santiago 7550000, Chile; juan.oyanedel@unab.cl; 4 Facultad de Economía y Negocios, Universidad Andres Bello, Santiago 7550000, Chile; andres.rubio@unab.cl; 5 Facultad de Psicología, Universidad Diego Portales, Santiago 8320000, Chile; 6 Escuela de Negocios, Universidad Adolfo Ibáñez, Santiago 7941169, Chile; wenceslao.unanue@uai.cl

**Keywords:** materialism at work, gratitude at work, longitudinal study, Chile

## Abstract

Materialism at work refers to a higher importance attached to extrinsic (e.g., money, fame, image) versus intrinsic (self-development, affiliation, community participation) employees’ ‘aspirations’. Research from self-determination theory has consistently found that materialism at work is strongly detrimental for both employees and organizations. For example, materialism is negatively associated with lower job satisfaction and engagement and positively associated with higher turnover intentions and job insecurity. Unfortunately, there are no viable strategies for reducing materialism in the workplace yet. In this sense, based on emergent research in psychology, we theorized that dispositional gratitude—a key construct within the Positive Organizational Psychology field—could be a protecting factor against materialism. Further, we conducted a three-wave longitudinal design among a large sample of Chilean workers (*n* = 1841) to test, for the first time, the longitudinal link between gratitude and materialism. We used two novel methodologies: A cross-lagged panel model (CLPM) to test between-person changes and a trait-state-occasion model (TSO) to test within-person changes. We found that both the CLPM as well as the TSO models showed that gratitude at work prospectively predicted further lower workplace materialism. Specifically, the CLPM shows that individuals with higher than average gratitude at Ti, are more likely to show lower than average materialism at Ti+1. The TSO shows that individuals with a higher than their usual level of gratitude at Ti are more likely to show a lower than their usual level of materialism at Ti+1. Important implications for materialism research as well as for the Positive Organizational Psychology field are discussed.

## 1. Introduction

Workplace materialism refers to a higher importance attached to extrinsic (e.g., money, fame, image) versus intrinsic (self-development, affiliation, community participation) aspirations at work [1,2]. Self Determination Theory (SDT; [3]) has consistently shown that materialism at work is associated with several undesirable outcomes (e.g., well-being, attitudes and behaviors) for both workers and organizations. For example, higher materialism decreases job satisfaction [1], organizational commitment [2], engagement [4], organizational citizenship behavior [5], and productivity [6]. Additionally, materialism increases turn over intentions, job insecurity and burnout [2], as well as workplace deviance [5], anti-ethical behaviors [7,8], and family-work conflicts [9]. Thus, how could the harmful effects of materialism be reduced is extremely urgent in order to protect employees’ well-being as well as company’s sustainability.

Unfortunately, there are no viable strategies for reducing materialism in the workplace yet. Researchers have focused on the negative consequences of materialism instead of its antecedents [10]. However, based on an emergent amount of studied in psychology, we theorized that gratitude—a key construct within positive psychology and positive organizational psychology [11,12,13]—might decrease workplace materialism. Indeed, a few scholars have found significant negative associations between gratitude and materialism in general life and educational settings [14,15,16,17], leading to hypothesize that it is very hard for a grateful person to be materialistic. On the one hand, grateful people strongly value and appreciate what they do have (e.g., family and social relationships) and want a better society (e.g., community involvement), which are all intrinsic aspirations. On the other hand, materialistic individuals feel envy for other people’s belongings, and put a strong emphasis on money and material possessions, which are all extrinsic aspirations. Further, we theorized that this association may also be found in the workplace: Increases in worker’s grateful disposition may decrease employee’s materialism.

To test our hypothesis, we conducted a three-waves longitudinal design, among a large sample of Chilean workers (*n* = 1841). Using novel methodologies, we tested a cross-lagged panel model (CLPM) as well as a trait-state-occasion model (TSO). Whereas a CLPM test between person differences, a TSO test within-person differences. Theoretical and practical implications might emerge from our research, especially for materialism research as well as for the Positive Organizational Psychology (POS) field.

### 1.1. Materialism at Work

Materialism, or the “importance ascribed to the ownership and acquisition of material goods in achieving major life goals” [18] (p. 210) has been conceptualized from multiple perspectives [19]. Initially, Belk [20] characterized the construct as personality traits (e.g., envy, non-generosity, and possessiveness) linked to the search for material possessions. Richins and Dawson [21] focused on personally internalized materialist values and beliefs. Kasser and Ryan [22,23] defined materialism in terms of the importance people attach to extrinsic (versus intrinsic) life goals and aspirations (see [10,24] for recent meta-analyses and reviews).

The Kasser and Ryan [22,23] approach is the main theoretical framework for studying materialism in mainstream psychology nowadays, and the one we used in the present research [24]. The authors developed the Aspirations Index (AI; [22,23]) to assesses the relative importance people attach to three extrinsic (e.g., fame, image, and wealth) versus three intrinsic (e.g., self-acceptance, affiliation, and community involvement) life goals. According to the authors, the higher the AI, the more materialistic a person is.

Research has consistently shown that higher materialism is strongly associated with lower well-being (e.g., life satisfaction, self-actualization, positive affect, vitality, and happiness) as well as with higher ill-being (e.g., negative affect, alcohol and substance use, physical symptoms, and depression [19,24]). The most accepted explanation for the negative link between materialism and well-being comes also from SDT [25,26]. SDT argue that the pursuit of intrinsic aspirations helps to fulfil three basic psychological needs (i.e., autonomy, competence and relatedness), that are essential nutrients for people quality of life, integration and flourishing. Thus, pursuing intrinsic aspiration foster psychological need satisfaction, which in turns increases well-being and decreases ill-being. However, pursuing extrinsic aspiration detracts people from satisfying the three basic psychological needs, thus decreasing well-being and increasing ill-being [3,19]. In other words, “whereas intrinsic goal pursuit may provide greater opportunities for need satisfaction, the pursuit of extrinsic goals may interfere with need satisfaction and even elicit experiences of need frustration” [19] (p. 571). Indeed, intrinsic aspirations are oriented towards self-actualization and self-expression, whereas extrinsic aspirations are focused on security and material acquisition [1].

The negative consequences of materialism extend to the workplace. Employees may pursue different kinds of aspirations. For example, whereas “*some employees view their jobs as opportunities to exercise their competencies and skills, pursue personal interests and make meaningful contributions to society*” (intrinsic aspirations) other employees may “*focus primarily on financial success, having control and influence over others and occupying a prestigious position at work*” (extrinsic aspirations) [1] (p. 251). Following Kasser and Ryan [22,23], the higher the relative importance workers attach to extrinsic (versus) intrinsic aspirations at work, the higher the workplace materialism.

Workplace materialism has been consistently associated with several undesirable outcomes for both employees and organizations in terms of well-being, attitudes and behaviors. For example, materialism relates to lower job satisfaction [1,27,28], organizational commitment [2,29], engagement [4,30], organizational citizenship behavior (OCB; [5,31]), mental health [32], self-esteem [33], and productivity [6,34]. Additionally, materialism also relates to higher turnover intentions and job insecurity [2], workplace deviance [5], burnout [35], anti-ethical behaviors [7,8], and family-work conflicts [9,36]. Thus, the negative consequences of materialism impact negatively not only worker’s personal life, but also employee’s mental health, well-being and performance, as well as organization′s sustainability. Further, findings mechanisms to decrease workplace materialism is urgently needed. This is the main goal of the present research, through the hypothesized role of dispositional gratitude.

### 1.2. Gratitude at Work

Gratitude has been conceptualized from several perspectives such as an emotion, a moral affect, a character strength, and a trait disposition (see [13,37,38,39] for reviews). Dispositional gratitude is the conceptualization we used in the present research.

Dispositional gratitude reflects to a “*wider life orientation towards noticing and appreciating the positive in the world*” [13] (p. 891). People high in grateful disposition show a higher tendency toward appreciating several abstract aspects of our daily life (e.g., a beautiful morning, a meaningful job, being alive). More grateful people feel gratitude for several aspects that humans tend to give for granted (e.g., been able to see, been able to walk, been able to breath), and thus experience gratitude with more frequency, intensity, and density, in comparison with less grateful individuals [13,17].

Research has found that gratitude relates to several indicators of well-being such as higher life satisfaction, positive affect/emotions, autonomy, competence, relationships, optimism, prosocial behaviour, and personal growth. Gratitude also relates to a multiplicity of indicators of ill-being such as lower negative affect/emotions, depression, anxiety, anger, and hostility [13,37,38,39,40].

Despite the extensive amount of gratitude research, studies in organizations are only in its infancy [41,42]. To date, only a few papers has shown the benefits of gratitude in the workplace. For example, gratitude at work has been associated with better organizational climate, enhanced employee’s well-being, and higher employee efficiency, success, productivity, and loyalty (see [41] for a review). Further, gratitude in organizations seems to “*appears to be a precious resource that sustains performance*” (p. 2).

Scholars have argued that the positive effect of workplace gratitude are due the construct is a kind of “antidote against toxic emotions at the workplace” [42] (p. 90). For example, more grateful employees tend to see their colleagues more positively, which in turns increases organizational citizenship behaviours (OCB), reciprocity, altruism, and foster high quality connection [41]. Importantly, gratitude may prevent against jealousy and envy, which used to be core elements of a materialistic way of living [20,42].

### 1.3. The Link between Materialism at Work and Gratitude at Work

Gratitude and materialism seem to be negatively correlated [14,15,16,17]. Two theorizations may explain the link. First, whereas grateful people are more intrinsically focused, materialistic individuals are more extrinsically oriented. On the one hand, grateful individuals highly value intrinsic aspirations such as helping others [43,44,45], building social relationships with other human beings [37,46,47], and a constant process of self-development [48]. Additionally, grateful people have a low interest in money and material possessions [17,49]. On the other hand, materialistic individuals strongly value extrinsic aspirations such as money, fame and image. Indeed, materialistic people focus on what they do not have instead of what they do have [16,17,50,51,52]. Because materialistic people are extrinsically focused, they are less likely to appreciate the good things in life, which is a key requirement for being a grateful person.

Second, the negative link between gratitude and materialism is rooted in the mediational role of life satisfaction [16]. Indeed, research has found that higher gratitude leads to higher life satisfaction, leading individuals to feel happy with their lives, detracting them of highly pursuing materialistic aspirations. Moreover, higher life satisfaction leads people to feel more security, which relates negatively to materialism [10]. Thus, higher gratitude leads to higher life satisfaction, which in turns increase the feeling of security, and thus decrease materialism [16,17,53,54].

Research has supported previous theorizations among students and youths. For example, McCullough et al. [17] found negative and significant correlations between gratitude and materialism. Lambert et al. [16] found that gratitude predicts lower materialism cross-sectionally and experimentally, and that life satisfaction mediated the association. Jiang et al. [15] and Chaplin et al. [14] found that gratitude was a protective factor against the dangers of materialism among adolescents.

Based on the previous evidence conducted mainly in general and educational settings, we theorize that gratitude negatively predicts materialism in the workplace. Grateful employees are more interested in helping colleagues in need, building strong social relationships with other employees, and developing its own skills (intrinsic aspirations). In addition, grateful workers are less interested in money, fame, and status (extrinsic aspirations) due they value what they have instead of wanting more material possessions. Thus, we expect that the higher the gratitude at work, the lower the workplace materialism.

### 1.4. The Present Study

As commented above, there is still a lack of longitudinal studies that confirm, as indicated in the literature, the prospective negative relationship between the dispositional gratitude of workers and materialism. Most prospective studies analyze between-person changes, while some others more related to personality variables deal with within-person changes [55]. However, the importance of combining both types of longitudinal analysis has been underscored recently, as this allows for understanding the relationship between the variables of a same individual across-time while observing the fluctuations between these variables in different individuals [56]. Dispositional gratitude is a variable studied as a trait, but people, in turn, work in organizations where they are constantly interacting with others. Thus, both levels of analysis should be considered. In this sense, we conducted a cross-lagged panel model (CLPM) to assesses between-person changes and an expanded multivariate latent trait-state-occasion model (TSO) to assesses within-person changes. Thus, based on our theoretical background, we have the following hypotheses:

**H1.**  *Gratitude at work prospectively predicts lower workplace materialism at the between-person level. In other words, we expect that if a person shows higher than average gratitude at work at Ti, she/he is likely to show lower than average materialism at work at Ti+1.*

**H2.**  *Gratitude at work prospectively predicts lower workplace materialism at the within-person level. In other words, we expect that if a person shows higher than their usual level of gratitude at work at Ti she/he is likely to show lower than their usual level of materialism at work at Ti+1.*

Although we theorized that gratitude predicts materialism, a few scholars have claimed that the reverse link may also be possible [57,58]. Thus, for the sake of comprehensibility and robustness methodological, we tested the following alternative hypotheses:

**H3.**  *Workplace materialism prospectively predicts lower gratitude at work at the between-person level. Further, we expect that if a person shows higher than average workplace materialism at Ti, she/he is likely to show lower than average gratitude at work at Ti+1.*

**H4.**  *Workplace materialism prospectively predicts lower gratitude at work at the within-person level. Further, we expect that if a person shows higher than their usual level of workplace materialism at Ti, she/he is likely to show lower than their usual level of gratitude at work at Ti+1.*

## 2. Method

### 2.1. Study Design

We conducted a three-wave longitudinal design, with three months between waves, among a sample of working adults over 18 years old in Chile. In order to test our hypotheses, we used two kinds of prospective analyses: We tested a CLPM as well as a TSO. Both the CLPM and the TSO need a large sample size. Thus, we aimed to recruit enough working adults to test a large (multiple latent variables across multiple time points) SEM (e.g., [59]).

### 2.2. Participants and Procedure

This research is part of a large project on happiness and well-being founded by the Chilean government. We followed standard ethical procedures of the American Psychological Association and the Declaration of Helsinki. Respondents were informed about the confidentiality of their data and their right to live the survey at any time without any penalty. The project was approved by the Ethics and Research Committee of a Chilean university. The Chilean university provided us with a list of working adults whose were sent an email containing and invitation to participate in a 3-waves longitudinal project, a brief description of the study, and a web-link to the questionnaire using QualtricsXM software (qualtrics, Provo, UT, USA). Only respondents who answer T1 survey were send further questionnaries three months (T2) and six months later (T3). Thus, we collected data for a full panel longitudinal design on three occasions over three months: Wave 1 (T1), Wave 2 (T2), and Wave 3 (T3).

At T1, 1841 (54.9% male; Mean age = 36.94, SD = 8.59) participants with ages ranging from 21 to 71 years successfully completed the survey. At T2, 979 participants (56.0% male; Mean age = 38.57, SD = 9.56) with ages ranging from 23 to 75 years completed the questionnaire. Finally, at T3, 700 Chilean workers (54.0% male; Mean age = 38.96, SD = 9.77) with ages ranging from 24 to 72 years completed the survey.

In terms of monthly income (measured in USD dollars at 26/03/2021), at T1 36.4% of workers had a salary lower than $1414.4 [T2: 31.7%; T3: 31.2%]; 42.4% had a salary between $1414.4 and $2828.3 [T2: 46%; T3: 46.5%]; 14.4% had a salary between $2828.3 and $4242.4 [T2: 14%; T3: 14.3%]; 4.2% had a salary between $4242.4 and $5373.8 [T2: 4.7%; T3: 4.5%]; and 2.6% had a salary higher than $5373.8 [T2: 3.6%; T3: 3.5%].

Regarding attrition [60], no statistically significant differences were found in terms of gender ([*χ*^2^ (2)] = 0.72, *p* = 0.070]), gratitude at work ([t(1839)] = −1.66, *p* = 0.097]) and materialism at work ([t(1839)] = 0.34, *p* = 0.073]) between those respondents whose left the survey after responding Wave 1 versus the rest of participants. We only found significant differences in terms of age ([t(686,63)] = −4.59, *p* < 0.001]). Little MCAR test [61] showed that missing data were completely at random ([*χ*^2^ (180)] = 205.030, *p* = 0.097]). Thus, we included all of the 1841 participants in our analyses, using full information maximum likelihood (FIML), to handle missing data [62].

Regarding normality, several authors agree that despite there is no clear rule for cut-off criteria, a conservative guideline assumes that multivariate normality should not be a problem if the threshold of the absolute values of skewness and kurtosis does not exceed 2 and 7 respectively [63,64,65]. Thus, Skew values for gratitude at work (T1: −0.33; T2: −0.42; T3: −0.42) and materialism at work (T1: −0.35; T2: −0.50; T3: −0.47) are acceptable in our data. Additionally, Kurtosis values for gratitude at work (T1: −0.24; T2: −0.05; T3: −0.28) and materialism at work (T1: 0.27; T2: 0.17; T3: 0.22) are also acceptable.

Regarding our sample size, we considered it is enough to test our SEM models appropriately. Indeed, it exceeds the ranges suggested for previous research. For example, Wolf, Harrington, Clarks and Miller [66] have claimed that a minimum of 30 and a maximum of 460 cases are sufficient. In addition, Weston and Gore [67] have shown that 10 cases per parameter do not generate improper estimates. Further, our sample size (*n* = 1841) fulfills previous requirements.

### 2.3. Measures

We used constructs with good psychometric properties. The original scales were translated using a standard back translation procedure [68].

#### 2.3.1. Materialism at Work

We used a short version of the Aspiration Index [23] adapted to the work context [69]. Participants responded on a scale from 1 (not at all) to 7 (very much) the importance they attach to extrinsic (fame, money and image) and intrinsic (self-development, community participation and affiliation) aspirations at work. Example items are “to have a job in which they are financially successful” (money) and “to have a job in which you can contribute to improving society” (community involvement). A workplace materialism index was build following a standard procedure [19] to rate the relative importance workers attach to extrinsic versus intrinsic life goals. First, we calculate a grand mean of aspirations including both the three extrinsic and the three intrinsic aspirations. Second, each aspiration was subtracted from the grand mean. Third, the three intrinsic aspirations were reversed. Fourth, and finally an overall extrinsic versus intrinsic (E/I) value score was computed by averaging the extrinsic and (reversed) intrinsic scales, where the higher the score, the greater the relative importance attached to extrinsic versus intrinsic workplace aspirations. We modelled materialism using three parcels. Each parcel was build using one extrinsic and one intrinsic (reversed) indicator [2,19,70]. Reliabilities measures using Cronbach’s alphas were good at T1 (0.71), T2 (0.73) and T3 (0.72).

#### 2.3.2. Gratitude at Work

We adapted the 6 items dispositional gratitude scale (GHQ-6) developed by McCullough et al. [17] to the work context. Participants responded on a scale from 1 (strongly disagree) to 6 (strongly agree) how much they agree or disagree with statements such as “I have too much to be thankful for in my work” and “As I get older, I become increasingly able to appreciate the people, events, and situations that are part of my work”. Reliabilities measures using Cronbach’s alphas were good at T1 (0.80), T2 (0.81) and T3 (0.80). We build a latent variable using the 6 indicators of the scale.

## 3. Results

### 3.1. Plan of Analysis

Table 1 shows descriptive statistics and zero-order correlations for all observed variables across waves. We used SEM and MPlus 8.0 [71] to test the structural relationships. Both the CLPM and the TSO were modelled using latent variables to reduce bias estimations.

Considering the benchmarks raised by Hu and Bentler [72], Kline [65] and Tabachnik and Fidell [73] we assess model fit through the following indicators: (1) RMSEA (root mean square error of approximation), (2) CFI (comparative fit index), (3) SRMR (standardized root mean square residual). According to the authors, acceptable model fit should fulfil the following standards: (1) RMSEA < 0.06; (2) CFI > 0.90 and (3) SRMR < 0.08.

### 3.2. CFA Analyses

We started with a confirmatory factor analysis (CFA) in order to examine the factorial validity of our core measures (gratitude at work and materialism at work) in each assessment time. At T1, results showed that the collapsed model (12 indicators; *χ*^2^ (54) = 2878.32, *p* < 0.001) is significantly worse than a model where gratitude (6 indicators) and materialism (6 indicators) were modeled as two different latent variables (*χ*^2^ (53) = 1734.58, *p* < 0.001), Δ*χ*^2^ (1) = 1143.74, *p* < 0.001. At T2, the collapsed model (*χ*^2^ (54) = 1556.93, *p* < 0.001) is significantly worse than the two-factor model (*χ*^2^ (53) = 1059.01, *p* < 0.001), Δ*χ*^2^ (1) = 497.92, *p* < 0.001. At T3, the collapsed model (*χ*^2^ (54) = 1073.39, *p* < 0.001) is significantly worse than the two-factor model (*χ*^2^ (53) = 808.711, *p* < 0.001), Δ*χ*^2^ (1) = 264.68, *p* < 0.001.

#### 3.2.1. Measurement Model

First, we set up a six-factor measurement model for gratitude and materialism. As suggested by Joreskog [74], we incorporated auto-correlated error terms for the observed indicators. We allowed all latent variables to covary freely across all time points. All factor loadings were significant (*p* < 0.001), and the model showed a good fit to the data, *χ*^2^ (282) = 1,356,155, *p* < 0.001, RMSEA = 0.045 (90% CI: [0.043, 0.048]), CFI = 0.91, SRMR = 0.07.

#### 3.2.2. Structural Models

The results of our CFAs analyses and the measurement model give us confidence in terms of the validity of our constructs in all waves. Thus, we proceeded to test the CLPM in Model 1 and the TSO in Model 2.

Model 1: Cross-lagged panel model

In our CLPM, each measure at (T+1) was regressed on its own lagged measure at (T) as well as on the other lagged measure at (T) [75,76]. Thus, all constructs were represented as potential antecedents and potential consequences of the other construct, while controlling for stability paths. Additionally, we allowed both gratitude at work and materialism at work to co-vary freely within each time point. First, we started with a 6-factor structural CLPM without any constrain. This model shows an acceptable fit *χ*^2^ (286) = 138,788, *p* < 0.001, RMSEA = 0.046 (90% CI: [0.043, 0.048]), CFI = 0.91, SRMR = 0.071. Second, to test invariance, we constrained all factor loading of each latent variable to be equal across waves. Model fit was also acceptable *χ*^2^ (300) = 140,334, *p* < 0.001, RMSEA = 0.045 (90% CI: [0.042, 0.047]), CFI = 0.91, SRMR = 0.073. Because the reduction in CFI is less than 0.01, the assumption of invariance was tenable (ΔCFI = 0.001; [77]), and thus was kept in our final model as well as in the TSO.

**Figure 1:**
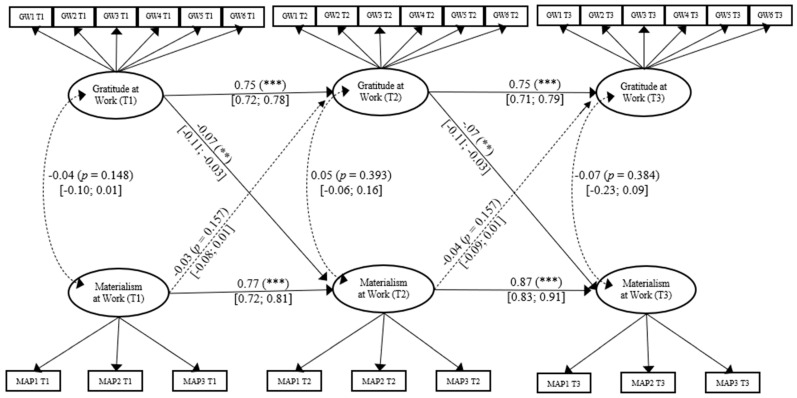
Model 1. Cross-lagged Panel Model for the Associations Between Materialism at Work and Gratitude at Work. *χ*^2^ (304) = 1410.816, *p* < 0.001; CFI = 0.91; RMSEA = 0.04. Note. All coefficients shown are standardized path. Error terms and factor loadings are not shown to enhance visual clarity. Loadings range all between 0.33 to 0.89 (*p* < 0.001). T1: Time 1; T2: Time 2; T3: Time 3. GWi: Gratitude at work item i. MAPi: Parcels materialism at work item i. Solid lines = significant paths, Dotted lines = no significant paths. Confident intervals are reported in square brackets. *** *p* < 0.001; ** *p* < 0.01; * *p* < 0.05.

Third, and finally, aiming for a more parsimonious model as well as to gain statistical power, we constrained all paths to be invariant over time [78]. This final model showed good fit, *χ*^2^ (304) = 141,082, *p* < 0.001, CFI = 0.91, SRMR = 0.074, RMSEA = 0.044 (90% CI: [0.042, 0.047]). Additionally, this model showed no significant differences compared with the previous model, Δ *χ*^2^ (4) = 7.47, *p* = 0.11. Factor loadings ranged from 0.33 to 0.89 (all *p* < 0.001). Supporting H1, gratitude at work was a significant negative predictor of materialism at work at the between-person level, *β* = −0.07, (95% CI. [−0.11, −0.03]), *p* = 0.001. However, H3 was not supported. Materialism at work did not predict gratitude at work, β = −0.03, (95% CI [−0.08, 0.01]), *p* = 0.16. No other significant paths were found (Because we constrained the paths from T1 to T2 to be equal to the paths from T2 to T3, for simplicity, we report here only the former. Our figures show all the details). The structural parameters are presented in Figure 1.

Model 2: Expanded Multivariate Latent Trait-State-Occasion Model

To complement the results obtained in CLMP model, we conducted a TSO, following Unanue, Martela, Vignoles, and Dittmar [79]. This methodology allows us to partition a latent variable (State) into two constructs, a completely stable latent variable (trait) and another latent variable that varies over time (occasion) [59,78,80]. This process allows us to estimate the within-person changes. First, we started modelling the state variables of gratitude at work and materialism at work as latent variables with factor loadings to each observed variable freely estimated auto-correlated uniquenesses for each observed indicator over time. Second, separate the state variance into a latent variable trait and occasion. Third, the latent variables of trait were loaded to the three state variables in their corresponding time with a fixed load of 1. Fourth, the occasion latent variables were loaded into the state variables in their respective time with a fixed load of 1. Fifth the residual variance of the state variables was set to zero. Sixth, covariances were estimated freely among the occasion variables within each time-point and among the trait variables. Seventh, we tested our hypothesis by modelling cross-lagged paths among the occasion variables following the recommendations of LaGrange et al. [59] that is to say each measure of occasion gratitude at work and materialism at work at (T+1) was regressed on its own lagged measure at (T) as well as on the other lagged measure at (T), Thus, all constructs were represented as potential antecedents and potential consequences of the other construct, while controlling trait-level relationships and stability across occasions.

We started with a 6-factor model with constrained loadings. This constrained model showed an acceptable fit, *χ*^2^ (297) = 1371.18, *p* < 0.001, CFI = 0.91 SRMR = 0.071, RMSEA = 0.044 (90% CI: [0.042, 0.047]). In second instance in order to increase the statistical power, we constrained all autoregressive and cross-lagged paths to be invariant over time [78]. This model showed an adequate fit, *χ*^2^ (301) = 1381.10, *p* < 0.001, CFI = 0.91, SRMR = 0.073. RMSEA = 0.044 (90% CI: [0.042, 0.047]) however, it differed significantly from the constrain model, (Δ.*χ*^2^ (4) = 9.92, *p* = 0.04). Inspection of all the respective paths found that the stability path of materialism needs to be estimated freely. This final model showed an adequate fit, *χ*^2^ (299) = 1374.45, *p* < 0.001, CFI = 0.91, SRMR = 0.073. RMSEA = 0.044 (90% CI: [0.042, 0.047]) and did not differed significantly from the original model, Δ *χ*^2^ (2) = 3.27, *p* = 0.19. Factor loadings ranged from 0.38 a 0.92 (all *p* < 0.001).

**Figure 2:**
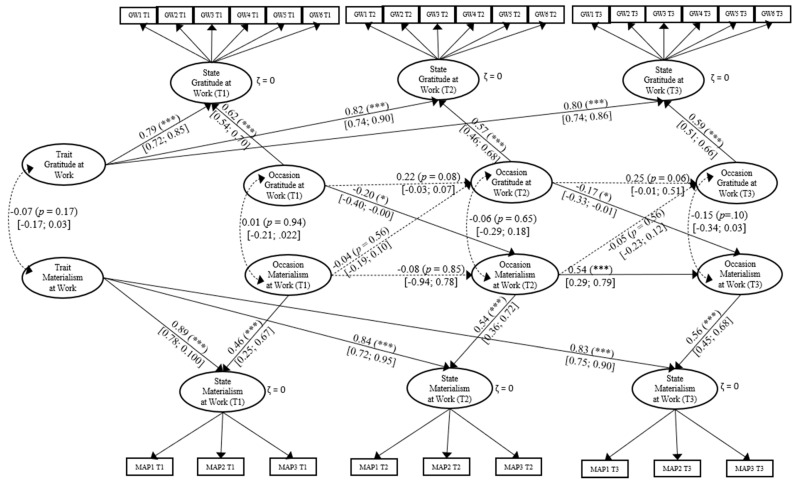
Model 2. Expanded Multivariate Trait-state-occasion (TSO) Model for the Associations Between Occasion Gratitude at Work and Occasion Materialism at Work. *χ*^2^ (299) = 1374.453, *p* < 0.001; CFI = 0.91; RMSEA = 0.04. Note. All coefficients shown are standardized path. Error terms and factor loadings are not shown to enhance visual clarity. Loadings range all between 0.38 to 0.92 (*p* < 0.001). T1: Time 1; T2: Time 2; T3: Time 3. GWi: Gratitude at work item i. MAPi: Parcels materialism at work item i. Solid lines = significant paths, Dotted lines = no significant paths. Confident intervals are reported in square brackets. *** *p* < 0.001; ** *p* < 0.01; * *p* < 0.05.

Based on this final model, we found that gratitude at work prospectively predicts lower materialism at work at the within-person level, *β* = −0.20, (95% CI. [−0.40, −0.00]), *p* < 0.01. Thus, H2 was supported. We did not find support for H4. Materialism at work was not a prospective predictor of gratitude at work, *β* = −0.04, (95% CI [−0.19, 0.10]). Details may be seen in Figure 2.

## 4. Discussion

SDT [3] has consistently shown that workplace materialism is strongly detrimental for both employees and organizations. Indeed, materialism at work has been associated with lower well-being and higher ill-being of employees, as well as with worse attitudes and behaviors. Therefore, studies like this one aim to find what factors can reduce materialistic aspirations at work. In this sense, the specific objective of this work was to determine the prospective relationship between gratitude at work and materialism, as well as confirming whether materialism is negatively and prospectively related to gratitude. 

Following our first and second hypotheses, prospective analyses were conducted considering the prospective relationship between gratitude and materialism based on between-person analysis (CLPM) and a within-person analysis (TSO). Concretely, these two types of analysis were carried out because both interpret the longitudinal relationships between the variables in different ways. For example, previous studies that used CLPM and TSO models have observed that sometimes these relationships can vary or even show opposite results depending on the type of analysis performed [81,82]. However, our study shows that gratitude at work predicts lower materialism at the between-person (CLPM) level, which is also true at the within-person level (TSO). This implies that, in the former case, individuals who scored high on the three measures across time showed a tendency to experience less materialism compared to other individuals. Therefore, this type of analysis allows for considering scores across time but always comparing them with other people in the sample. As for TSO, the results indicate that gratitude influences materialism prospectively at the level of individual workers. Within-person analyses are especially relevant when constructs that behave as attitudes or stable traits are considered independent variables, and therefore this type of analysis allows for determining how these traits affect other variables across time [83].

In summary, the negative relationships observed in both types of longitudinal analysis between gratitude and materialism suggest that interventions and programs oriented to increase gratitude in work contexts could promote a decrease in this type of aspirations. According to the previous literature, gratitude is an emotion that from an evolutional perspective, contributes to better interpersonal relationships that are stable over time because gratitude is strongly related to prosociality [17,84]. This is a key aspect to understand the negative relationship with materialism, as people with high gratitude are more oriented to others instead of themselves, which promotes altruistic and prosocial behavior towards others [43]. Conversely, materialistic people are characterized by pursuing fame and image, and therefore their aspirations seek self-glorification [1,2]. In the workplace, the constant search for a higher salary, more social prestige, etc., can end up generating less happiness and more negative affect than others [69,85]. While the constant experience of gratitude may contribute to better work relationships with coworkers, a higher capacity to deal with daily problems and consequently more personal resources to face challenges at work [86]. For example, in a recent 3-week gratitude intervention with 835 employees conducted by Komase et al. [87], personal resources such as self-efficacy and job performance improved considerably, whereas psychological distress decreased.

Hypotheses 3 and 4 aimed to determine whether materialism can also explain prospectively and negatively gratitude in workers. This aspect should be considered as aspirations are long-term objectives that directly influence our choices and lifestyles [3,88]. In this sense, since materialistic people usually are focused on extrinsic aspirations such as money, fame and image (e.g., [16,52], this might affect the experience of gratitude and materialism, in turn, would influence gratitude prospectively. However, both the between-person analysis (CLPM) and the within-person analysis (TSO) did not show significant negative relationships between both variables across time. These *a priori* results may seem surprising as materialism, in addition to being negatively related to different work well-being, is also often associated with lower-quality relationships [5,89]. Materialistic people, in turn, focus more on their own benefits and this makes it more difficult for them to center on the needs of others, or to experience more prosocial behavior towards other individuals [26,90]. However, in this study materialism at work has been specifically associated with the experience of gratitude in the workplace. The relationship is also negative but not significant across time when considering both levels of analysis. This poses new questions about whether intrinsic aspirations in the workplace can promote the experience of gratitude as opposed to materialism. 

The results show that gratitude can contribute to a decrease in the materialism of workers across time but delving into the variables may promote that these workers experience more gratitude at their positions. As indicated by previous studies, gratitude at work can be increased through programs and interventions, but it is necessary to understand if that trait can be developed and then maintained over time. Thus, more longitudinal studies are required on this specific topic.

### 4.1. Theoretical Implications

First, it remains to be demonstrated whether the impact of gratitude on materialism have the same effects in an unexplored and specific domain: The workplace. To date, research has been conducted only in general life settings or in specific domains such as schools [14]. Thus, we tested for the first time the link between gratitude and materialism in organizations. Second, we used sophisticated methods to test the longitudinal link between gratitude and materialism. Our CLPM and TSO models allowed us not only to explore the prospective associations between the constructs, but also the directionality between them, as well as how changes in gratitude may lead to changes in materialism over time. Third, we are the first to date exploring a likely protective factor against the multiplicity of negative effects of materialism at work (i.e., gratitude). We think it is our most novel contribution.

### 4.2. Practical Implications

We hope our results may help academics and practitioners in the Positive Organization Psychology field (POS) to find new ways of improving workers’ quality of life, as well as building healthier and more resilient organizations [91]. Indeed, because we found that gratitude may decrease materialism prospectively, employees, companies and practitioners may use a broad set of gratitude interventions in order to decrease this toxic workplace attitude, which in turns may protect employees’ mental health as well as a company’s sustainability. In addition to finding new ways to fostering gratitude at work, we also encourage workers and organizations to decrease materialism directly in the work place.

#### 4.2.1. Fostering Gratitude at Work: Challenges for Employees and Organizations

Positive psychology as well as the POS field has developed outstanding strategies to increase gratitude in organizations. In the present section, we will outline three of them. First, following Seligman et al. [92] we recommend using the gratitude letter exercise recommended for the organization context by Salanova et al. [91]. The authors proposed that workers write a letter of thanks or an email in order to deliver it to a person who has been really kind and special to him/her, but who has never been recognized appropriately. Research has found that participants who followed this exercise, not only increased their gratitude in comparison with the control group and those who received a placebo, but also improved several indicators of well-being [92]. In order to ensure that the beneficial effects of the exercise last long enough, both activities must be developed in combination, i.e., write the letter and deliver the letter [93]. This exercise, ideally, should be spread across the whole organization.

Second, following Emmons and McCullough [94] organizations may recommend workers to follow regularly (e.g., every week) the practice of the so-called “three good things” exercise [92]. For instance, before going to sleep employees write “three good things” that happened to them in their workday [91,94]. Research has found that people who follow this “gratitude journal” practice (i.e., counting their blessings) showed an increase in gratitude, positive states of alertness, enthusiasm, determination, attention, energy, prosocial behavior, and life satisfaction compared to control or hassles conditions. [92,94].

Third, and finally, Fehr et al. [95] developed a multilevel model of gratitude “*composed of episodic gratitude at the event level, persistent gratitude at the individual level, and collective gratitude at the organizational level*” in order to build a “*grateful workplace*” (p. 361). The authors strongly recommend that organizations develop formal and measurable gratitude programs. Among some practices, Fehr and colleagues suggested developing appreciation programs, beneficiary contact exercises, developmental feed-back programs, human resources’ alignment, and closer contact between employees and customers (among others), which may foster the three kinds of gratitude already mentioned, but also increases workers well-being and attitudes, as well as a company’s performance.

#### 4.2.2. Decreasing Materialism in the Workplace: Challenges for Employees, Leaders and Organizations

Strategies aimed to decrease materialism should focus on (a) decreasing the importance attached to extrinsic goals, (b) increasing the importance attached to intrinsic goals, or a combination of (a) and (b). These strategies are not easy at all, because we live in a consumer culture [24] that shows us every day—erroneously—that money and possessions are the pathway to happiness and well-being. Nonetheless, there are still key actions that workers and organizations may pursue in order to protect employees’ mental health and companies’ sustainability from the dangers of materialism.

Science has shown that extrinsic and intrinsic goals are a kind of “see-saw” [96]. For example, when extrinsic goals go up, intrinsic goals tend to go down. This helps to explain why people act in less cooperative and generous ways in the workplace when salary and pay rises are their priorities. Fortunately, when employees focus on intrinsic goals such as helping others and building strong ties, the importance attached to money, fame and image tend to decrease [96]. Based on the previous evidence, first, we strongly encourage workers to attach a high importance to intrinsic work goals. By doing this, the importance attached to extrinsic goals will almost automatically decrease, thus lowering the individual’s materialism. Second, the reverse strategy is also important. Indeed, we also encourage workers to attach a low importance to extrinsic work goals. By doing this, the importance attached to intrinsic goals will almost automatically increase, thus lowering employee’s materialism.

Research has also shown that materialism increases when people feel insecure and have worries about their self-worth [96,97]. When this happens, people tend to focus on extrinsic aspirations as coping strategies. Thus, researchers recommend that instead of searching for more money, fame and image (as an extrinsic coping strategy), workers should try to move toward intrinsic coping strategies. For example, by building strong social relationships with their colleagues, by helping other employees in need, and/or by growing as a person, employees may reduce their own materialism. Finding meaningful work and experiencing engagement has also been proposed as other fruitful strategies to promote intrinsic goals, thus protecting employees against the dangers of materialism [2].

Previous recommendations point toward a change in employee’s life style. However, leaders and companies also have a big responsibility in decreasing materialism at work. Indeed, it has been argued that materialism is present in the value system of several organizations, due to strongly encouraging the pursuit of extrinsic goals such as productivity, efficiency and profitability despite the costs it has on worker´s quality of life [98,99]. Unfortunately, given the materialistic focus of some companies, “non-materialistic individuals are unlikely to be attracted to these materialistic organizations and choose to work elsewhere” [27] (p. 1021); [100]. Thus, companies with a materialistic focus may not be able to hire highly skilled non-materialistic individuals, which may affect a company’s performance. Further, three strategies may be followed by leaders and organizations in order to protect employees’ well-being and companies against materialism.

First, leaders and organizations should develop formal policies to encourage the pursuit of intrinsic values (e.g., corporate altruism and self-development). Second, it has been shown that exposing workers to materialistic messages could increase the pursuit of extrinsic values, which in turn may increase workplace materialism [99,101]. Further, whereas the importance attached to pay systems, bonus and performance ratings should be reduced, the importance attached to employee´s intrinsic motivation, well-being and mental health should be increased by leaders and organizations [99]. Third, a higher importance attached to work-family balance, responsible time use as well as to compassion and transparency across the whole organization are key elements for decreasing workplace materialism [102].

## 5. Limitations

Like all kinds of research, we recognize that we also have some limitations. First, the inclusion of non-self-reported measures is an important element that should be considered in future studies. However, to date, the most stablished method to capture gratitude and materialism is through self-reporting. [24,38]. Additionally, McCullough et al. [17] showed that there were no significant differences between self-report and third-part reports of gratitude, which make our method more reliable.

Second, although our study helps to estimate the prospective relationships between gratitude and materialism, it does not exclude the option of third variables excluded (e.g., omitted variables) that may be playing a role. Third, despite the fact that prospective/longitudinal associations are a key requirement for establishing causality, this kind of design cannot test causal associations properly. Thus, we encourage further research to conduct experimental designs. Fourth, our sample does not allow cross-cultural generalizations. Future research should explore different context and cultures. Nonetheless, our research in Chile advanced significantly the existing knowledge regarding the studied variables. Fifth, we collected demographic data only for age, gender, and salary. That is because we were interested in studying working adults in general, without any restrictions. Nonetheless, we recognize that it would be important to have more information about a participant´s profile such as the kind of company they work for (mining, sales, etc.); whether or not participants work for public or private organizations; whether or not they have managerial responsibilities and the specific job area of the participants (e.g., marketing, finance, operations, etc.). Sixth, and finally, it would be important to reduce attrition rates in future research. However, this issue is common when using longitudinal designs [13,103]. Fortunately, Little’s test showed that missing data was completely at random, which allowed us to use FIML estimation through modern imputation procedures.

## 6. Conclusions

The results show negative prospective relationships between gratitude at work and workplace materialism considering two levels of analysis, namely between- and within-person. This implies that people with high gratitude at work exhibit less materialism across time. In turn, people who score high on gratitude at work also show lower levels of materialism than their peers. These results reinforce the relevance of programs and interventions aimed to foster gratitude at work in order to reduce the materialistic aspirations of workers. On many occasions, organizations promote reward systems that focus on success and prestige at work. However, according to the literature, this has been related to lower levels of work satisfaction and a higher perception of work overload. Concretely, materialistic people tend to be more focused on strengthening their fame and image, and consequently their own self, whereas gratitude promotes better interpersonal relationships and is an emotion centered on collective well-being. In this sense, the results of this study show that gratitude can reduce the materialism of workers across time. Nevertheless, further studies are necessary to delve into the prospective relationship between the gratitude of workers and other variables associated both to organizational climate and the welfare of workers.

## Figures and Tables

**Table 1:** Descriptives and Zero-order Correlations Between all Variables at T1, T2 and T3.

	M	SD	1	2	3	4	5	6
1. Gratitude at Work T1	5.09	1.14	1					
2. Gratitude at Work T2	5.15	1.13	0.67 **	1				
3. Gratitude at Work T3	5.22	1.11	0.65 **	0.67 **	1			
4. Materilalism at Work T1	3.02	0.70	−0.11 **	−0.08 **	−0.13 **	1		
5. Materilalism at Work T2	3.03	0.70	−0.16 **	−0.11 **	−0.16 **	0.63 **	1	
6. Materilalism at Work T3	2.97	0.70	−0.20 **	−0.18 **	−0.23 **	0.60 **	0.71 **	1

*Note*. T1: *n* = 1841; T2: *n* = 979; T3: *n* = 700. T1: Time 1; T2: Time 2; T3: Time 3. ** *p* < 0.01.

## Data Availability

Not applicable.
